# Case Report: Pseudoprogression With Nivolumab and Bevacizumab Followed by Recurrent Immune-Related Pneumonitis in Urothelial Carcinoma With Lung Metastasis

**DOI:** 10.3389/fonc.2020.611810

**Published:** 2021-02-02

**Authors:** Zizhong Yang, Guoqing Zhang, Qiong Sun, Minglu Liu, Jiakang Shao, Shunchang Jiao

**Affiliations:** ^1^ Department of Oncology, Chinese People's Liberation Army (PLA) General Hospital, Beijing, China; ^2^ School of Medicine, Nankai University, Tianjin, China

**Keywords:** pseudoprogression, urothelial carcinoma, immune-related pneumonitis, immune checkpoint inhibitor, anti-angiogenesis, case report

## Abstract

**Background:**

Combination therapy with immune checkpoint inhibitors (ICIs) and antiangiogenic agents is generally effective and well tolerated and might be effective for metastatic urothelial carcinoma (UC). However, ICI treatment is often associated with unique responses, such as pseudoprogression and ICI-related pneumonitis (CIP), which may influence clinical decision making and affect treatment. Although there have been many studies on the mechanism of pseudoprogression and CIP, the characteristics and relationship of these special events in a clinical setting remain rarely reported.

**Case Presentation:**

Here, we present a patient with lung metastatic UC who underwent surgery and two lines of chemotherapy. The programmed cell death-1 (PD-1) inhibitor nivolumab and antiangiogenics agent bevacizumab were used as maintenance treatments. The patient experienced pseudoprogression after 2 PD-1 inhibitor cycles. The lesions in both lungs were enlarged on computed tomography (CT) imaging, and treatments were continued for another two cycles, after which the tumor size decreased to below baseline, followed by a durable response. However, after 4 months of pseudoprogression, the patient then developed CIP. The CIP was responsive to glucocorticoid therapy but recurred during ICI rechallenge, leading to the termination of immune therapy. Ultimately, the patient achieved durable, stable disease for over 18 months without further anticancer treatment.

**Conclusions:**

Our case shows that pseudoprogression can occur in UC during immunotherapy even when combined with an effective antiangiogenic agent. In addition, pseudoprogression may be correlated with future adverse effects and a durable response. In the management of CIP, early rechallenge with ICIs may lead to CIP recurrence, which could be more severe and needs to be treated early and with appropriate drugs. Clinicians should be aware of atypical responses to ICIs and adjust the treatment plan accordingly.

## Introduction

Immune checkpoint inhibitors (ICIs), such as nivolumab, have revolutionized the treatment of cancer. They can enhance the antitumor immune response by T cells by targeting programmed cell death-1 (PD-1) or cytotoxic T-lymphocyte-associated protein 4 (CTLA4). In the clinical trial Checkmate 275 (1), a median survival time of 8.74 months was achieved for patients with recurrent and unresectable urothelial carcinoma (UC) treated with nivolumab, which was significantly superior to cytotoxic treatment. Nivolumab has been approved as the first-line treatment for advanced-stage UC.

One treatment phenomenon that is increasingly recognized in the era of immune therapy is pseudoprogression. Pseudoprogression is an atypical response of solid tumors under treatment with an ICI and is characterized by transient increases and subsequent decreases in the volumes and number of lesions. According to a previous study, UC has an incidence of pseudoprogression ranging from 1.5% to 17% (2). The pseudoprogression shows various abnormalities by radiography and is often misdiagnosed as real disease progression (3).

Current research on pseudoprogression has focused on ICI monotherapy or ICI combined with cytotoxic drugs. In recent years, ICI treatment has often been combined with antiangiogenic agents. Antiangiogenic agents can increase vessel permeability and promote tumor antigen expression. They change the pattern of the immune response. However, pseudoprogression during combination treatment with ICIs and antiangiogenic agents has rarely been reported (4). Whether antiangiogenic agents influence the incidence or characteristics of pseudoprogression during ICI treatment remains unknown.

Another unique effect of ICI treatment is ICI-related pneumonitis (CIP). CIP is considered to be an interstitial lung injury caused by the overactivation of the immune system. CIP is rare but severe. The overall incidence of CIP in PD-1 monotherapy is only approximately 2.7%, but approximately one-third of CIP patients develop severe (Common Terminology Criteria for Adverse Events (CTCAE) grade 3 or higher) symptoms (5). Although most CIP cases are steroid responsive, ICI treatment needs to be suspended when patients develop grade 2~4 CIP, thus increasing the risk of tumor progression. Rechallenge with immunotherapy after CIP has become a major problem. According to the guidelines (6), restarting ICI treatment may increase the recurrence rate of CIP. However, the clinical manifestations and outcome of recurrent CIP have rarely been reported.

**Figure 1:**
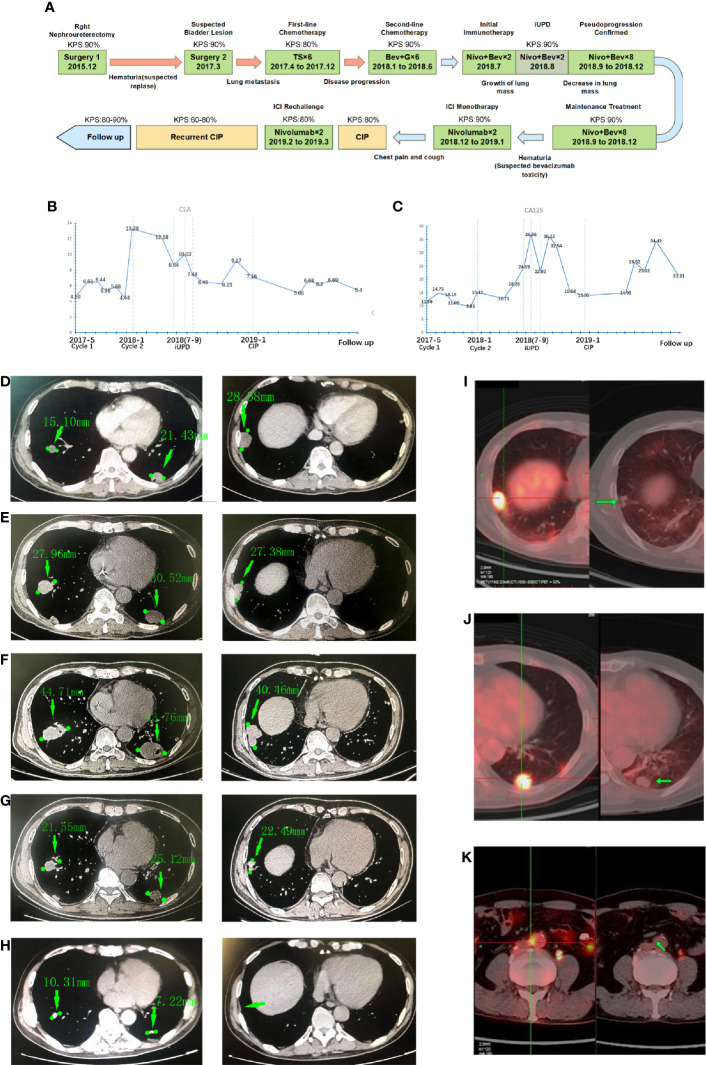
The time line, biomarker and medical imaging information of the patient. **(A)** Time axis of antitumor treatment and intervention in terms of side effects. (T = paclitaxel; S = S-1; G = gemcitabine; Bev = bevacizumab; Nivo = nivolumab), Karnofsky Performance Status (KPS) refer to the Karnofsky performance status during that period. **(B)** Carcinoembryonic antigen (CEA) levels during treatment; **(C)** CA125 levels during treatment. Chest CT images showing the tumors on lungs: **(D)** The initial images at the time of diagnosis showed multiple pulmonary metastases and bronchial obstruction; **(E)** The images of the same lesion before the immunotherapy; **(F)** The pulmonary metastases were enlarged transiently on the first follow-up examination; **(G)** The lesion was decreased after the subsequent two cycles of treatment; **(H)** Further tumor shrinking on the latest follow-up images, the lesion in right inferior lobar almost disappeared. Positron emission tomography (PET)/computed tomography (CT) of the patient: The left panel of each picture shows the initial image of the lung metastases, and the right panel shows the images of the same lesions 10 months after the termination of immune checkpoint inhibitor (ICI) treatment: **(I)** Metastatic tumor in the right lung; **(J)** Metastatic tumor in the left lung; **(K)** Paraaortic node metastases and lesions disappeared after treatment.

In this article, we present a rare case of lung metastatic UC treated with ICI (**Figure 1A**). To our knowledge, this is the first report of UC pseudoprogression during combination ICI and antiangiogenic therapy. Moreover, this patient also experienced late-onset recurrent CIP during further treatment with nivolumab, while the tumor remained continuously stable. Using this case, we review the characteristics of some special manifestations or adverse effects of immunotherapy that may affect clinical decisions.

## Case Presentation

In December 2015, a 52-year-old man was admitted to our hospital when hematuria prompted an intravenous pyelography (IVP) and computed tomography (CT) evaluation that revealed a distinct right kidney mass. The patient was in good condition (Karnofsky Performance Status (KPS) = 90%) and complained of mild back pain without dysuria or weight loss. He has concomitant anemia (grade 1) and chronic kidney disease (CKD grade 2), receiving benazepril with low-protein diets to control proteinuria and calcitriol to prevent osteoporosis; no other conditions (including diabetes, hypertension, pulmonary or autoimmune diseases); a history of tobacco use for 20 years; and no history of alcohol or drug abuse. Physical examination was notable for percussive pain in the right renal region and no palpable mass; the remainder of examinations were normal. Blood examination showed that the hemoglobin was 125 g/L, serum creatinine was 115.2 µmol/L, electrolytes and liver function were generally normal (**Supplemental Table 1**).

He underwent total right nephroureterectomy. Pathology from resection was consistent with UC (T2N0M0). In March 2017, a regular cystoscope reexamination revealed a mass in the bladder that was considered UC recurrence. The patient then underwent another surgery to remove that lesion. One month after the second surgery, he presented with hoarseness and cough. A CT scan of the chest revealed multiple nodules in the posterior basal segment of both lungs consistent with metastatic tumors. In May 2017, the patient received a percutaneous biopsy for lung lesions, and the immunohistochemical staining results confirmed metastatic tumors of urothelial origin (ALK (Ventana) (-), CK7 (+), Napsin A (+), p63 (+), TTF-1 (-), CK5 (+), p40 (+), GATA3 (+), and PD-L1 (50% tumor cells)). Further positron emission tomography (PET) showed tumors in the lungs and paraaortic lymph nodes (**Figures 1D, I–K**).

From May 2017 to December 2017, he received six cycles of paclitaxel (175 mg/m^2^ d1) + S-1 (a novel 5-fluorouracil-based agent containing tegafur, gimeracil, and oteracil) (60 mg/day d1 to d14) every 21 days as first-line chemotherapy, granisetron and dexamethasone were also used for emesis controlling. The patient developed fatigue, anorexia, and hair loss during the treatment. The myelosuppression culminated after four cycles with leukocyte fell to 2.76*10^9^/L and erythrocyte to 3.68*10^12^/L. Erythropoietin, granulocyte colony-stimulating factor (G-CSF) and leucogen were used to promote hematopoiesis. In January 2018, lung progression occurred on CT, and the patient started second-line treatment with gemcitabine (1,000 mg/m^2^ d1 and d8) + bevacizumab (5 mg/kg d1) in a 21-day cycle. After another six cycles of treatment, the patient had a better performance status and stable disease of his lungs (**Figure 1E**) and urinary tract.

Owing to the potential nephrotoxicity of gemcitabine, chemotherapy was stopped and changed to maintenance of immunotherapy. As PD-L1 was highly expressed in the tumor, in July 2018, he started treatment with the PD-1 inhibitors nivolumab (140 mg) and bevacizumab (300 mg) every two weeks. The treatment process went smoothly with no sign of progression. However, in August 2018 (at the evaluation after two cycles), the CT scan revealed that the lesions in the lungs were significantly enlarged (largest lesion from baseline 30 mm to 43 mm, **Figure 1F**) with elevated tumor biomarkers cancer antigen (CA) 125 and carcinoembryonic antigen (CEA) (**Figures 1B, C**), while the patient had no obvious symptoms or signs. This was consistent with the unconfirmed progressive disease (PD) criterion in immune Response Evaluation Criteria in Solid Tumors (iRECIST). Considering their potential survival benefit to the patient, nivolumab and bevacizumab treatment was continued. On the next follow-up CT (6 weeks after sustained treatment), the sizes of the tumors in the lungs had markedly decreased (**Figure 1G**), and his residual lesion in paraaortic node disappeared. Over the next eight cycles of treatment, the patient did well, with a durable response and no tumor progression.

In December 2018, the patient developed intermittent macroscopic hematuria (CTCAE-grade 1) again after the 12th treatment. A CT scan with contrast confirmed that the ureter remnant was stable with no evidence of a new tumor. According to previous studies, a common toxicity of bevacizumab is bleeding (7). The hematuria was likely related to bevacizumab. The treatment was changed to nivolumab monotherapy, and hematuria was relieved soon afterwards.

**Figure 2:**
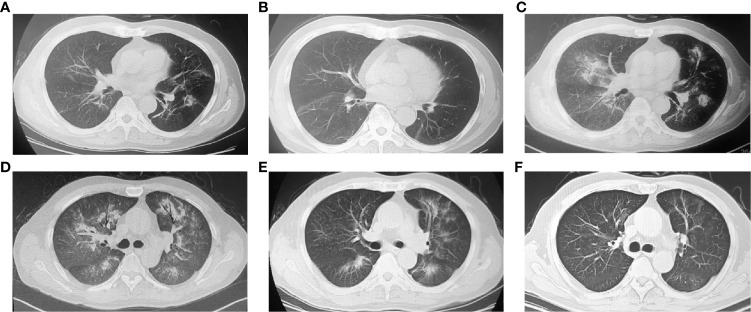
Chest computed tomography (CT) images showing the ICI-related pneumonitis (CIP) **(A)** ground-glass opacity in both lungs. **(B)** Inflammation absorbed after 2 weeks of glucocorticoid treatment. **(C)** CIP recurrence after resuming nivolumab treatment. Ground-glass opacity (GGO) reoccurred, and more lobes of the left lung were involved. **(D)** During the period of recurrent CIP, both lungs showed diffuse GGO, consolidation and the air bronchus-charging sign. **(E)** Image after 7 days of corticosteroid pulse therapy. The extent of CIP was reduced. **(F)** Image after 4 months of CIP treatment. The signs of pneumonitis subsided.

In January 2019, the patient developed cough, shortness of breath and chest pain without fever or expectoration. Chest radiography revealed diffuse ground-glass opacity (GGO) in both lungs without progression of metastatic tumor lesions (**Figure 2A**). The blood test showed C-reactive protein and procalcitonin were normal, and there were no pathogenic bacteria in sputum culture (**Supplemental Table 1**). Pulmonary infection was also excluded. Based on his symptoms, imaging features and treatment history, CIP (CTCAE-grade 2) was diagnosed. ICI treatment was immediately stopped, and he received intravenous methylprednisolone (2 mg/kg) for 3 days. The symptoms and radiography showed obvious improvement (**Figure 2B**).

The steroid was then changed to oral methylprednisolone and tapered over 1 month. Nivolumab (same dose) was restarted 2 weeks later. However, on March 2019 (after two cycles of nivolumab treatment), the patient was admitted to the hospital again with worsening shortness of breath (KPS = 60%). Radiography showed more GGO in the lungs (**Figure 2C**), and the percutaneous oxygen saturation (SpO2) was 93%. Nivolumab treatment was soon terminated, and methylprednisolone (2 mg/kg iv) was reused to control inflammation. The treatment was effective at first. The symptoms were partially controlled after 7 days of methylprednisolone. However, the consolidation in the lungs was not improved. Two weeks later, the patient’s symptoms were aggravated again, and pneumonitis progressed in multiple lobes (**Figure 2D**). CIP (CTCAE-grade 3) was diagnosed. The patient received treatment with a larger dose of methylprednisolone (4 mg/kg iv.) with pirfenidone and broad-spectrum antibiotics (patient was CKD3 at that time, so immunosuppressors such as cyclophosphamide were not considered). His symptoms improved gradually after 8 weeks of steroid treatment, and he ultimately recovered (**Figures 2E, F**).

During follow-up over the next 4 months of CIP treatment without ICI, the tumor remained stable. Surprisingly, a PET scan in June 2019 revealed that the tumors in the lungs and paraaortic lymph nodes were reduced with no abnormal metabolic activity (**Figures 1I–K**). After that, the patient was regularly seen for follow-up every 3 months without further antineoplastic treatment. He was generally in good clinical condition (KPS 80-90%) without the onset of new relevant symptoms. The last CT scans were performed in September 2020, and there was still no sign of tumor progression (**Figure 1H**).

## Discussion

Pseudoprogression is an unusual response of tumors. It is believed that pseudoprogression is an ICI-induced infiltration of lymphocytes into tumors or the edema and necrosis of tumor tissue after therapy rather than real tumor growth. Pseudoprogression is common in glioblastoma, lung cancer, and melanoma but has been rarely reported in UC. Despite having a more favorable effect than conventional ICI monotherapy, the pseudoprogression of combination ICIs and antiangiogenic agents seems to be rare. Our patient had metastatic UC. His disease was stable with bevacizumab combination chemotherapy, but he developed pseudoprogression after switching to nivolumab plus bevacizumab treatment, which has never been reported before. This result indicated that pseudoprogression could still occur in a patient with ICIs plus antiangiogenic agents, even though the antiangiogenic agent was previously effective.

Histopathologic examination was the only way to confirm pseudoprogression. However, in clinical practice, second biopsies are difficult to obtain. A practical method for the clinical diagnosis of pseudoprogression is needed. In recent years, new imaging examinations, such as PET and functional magnetic resonance imaging (MRI) have been shown to have advantages in pseudoprogression diagnosis by detecting the metabolism or specific macrophages in tumors. The value of biomarkers has also been emphasized, and Zhou’s study in non-small-cell lung cancer (NSCLC) showed that if CA125, CA199, or CEA increase by less than 50% above baseline after immunotherapy, it could still indicate the disease control (8). This is in agreement with the biomarker variation in our case.

According to a recent meta-analysis, approximately 2.2% of patients develop CIP during nivolumab treatment (7). Most CIP occurred at the initiation of immunotherapy, with a median time of 2.8 months. However, in our patient, CIP occurred 6 months after ICI treatment. In the treatment of CIP, most patients with grade 1 to 2 CIP were sensitive to corticosteroid treatment (9). This was in line with our study in which initial CIP rapidly resolved after pulsed methylprednisolone. ICI treatment may be continued when CIP downgrades to grade 1 or less, but there is no consensus on the strategy for ICI rechallenge. In our patient, we fully restored the nivolumab treatment 2 weeks after the CIP-associated changes completely resolved, but severe CIP still recurred. In addition, the recurrent CIP was more severe in regard to symptoms and imaging features with less sensitivity to glucocorticoids, which differed from previous studies. Therefore, we suggested that ICI reintroduction needs to occur slowly and cautiously. To prevent fatal side effects, only patients with good clinical condition (KPS > 70%) should be considered. If CIP recurs, the ICI needs to be withdrawn immediately. Recurrent CIP should be treated early with appropriate drugs, including higher levels of glucocorticoids, and tapered slowly to ensure that inflammation is controlled.

In our case, the patient developed CIP approximately 4 months after pseudoprogression, which indicated that pseudoprogression may be relevant to subsequent immune-related adverse events (irAEs), including CIP. Interestingly, Kim et al. reported a pseudoprogression case of UC treated with durvalumab. This patient had pseudoprogression that manifested as pneumonitis-like GGO around lung metastatic lesions, where CIP could not be excluded (3). According to previous studies, both irAEs and pseudoprogression could be regarded as evidence of rigorous immune activation that correlate with survival benefits. Hui et al. had used early-onset irAEs as a predictor to differentiate pseudoprogression (10). Alternatively, as another sign of immune overactivation, pseudoprogression may also predict future irAEs. It has clinical value for irAEs prediction, especially in the case of durable ICI responses. If a patient develops pseudoprogression, the clinicians also need to pay attention to the risk of immune-related adverse reactions in the future. However, many other drugs have pulmonary toxicity, and there were also reports of paclitaxel- or bevacizumab-induced pneumonitis (11), although our patient did not experience any respiratory adverse reactions during treatment before ICIs. Their effect in the occurrence of CIP needs further investigation.

Continuous ICI treatment can generate specific antitumor immunity by relieving the tumor’s immune inhibition. Clinical trials including CheckMate 032 (12) have revealed that patients with long-term ICI treatment may generate a durable immune response. In our case, after 12 cycles of ICI treatment with a series of adverse events, the patient achieved stable disease for more than 33 months of follow up. This led us to prefer to maintain ICI treatment for sensitive patients, including after pseudoprogression. However, prolonged treatment also increased the risk of irAEs. This highlights the necessity of achieving long-term survival benefits and mitigating the dose-dependent side effects of ICIs. Moreover, Christiansen’s study showed that melanoma patients who discontinued nivolumab due to toxicity had long off-treatment survival without progression (13). In our case, the ICI was terminated due to severe CIP, but the disease remained stable. Although PET/CT examination showed old lesions, no abnormal uptake was found, suggesting that the lesions were inactive.

In conclusion, this is the first case of pseudoprogression in UC during combination ICI and antiangiogenic therapy, and we summarized some characteristics and the relationship of two ICI-related atypical responses (pseudoprogression and CIP) in this rare patient. The occurrence of pseudoprogression during ICI treatment might be correlated with future irAEs and a durable response. ICI reintroduction after CIP needs to be slowly, as it may lead to severe CIP recurrence. The treatment of recurrent CIP needs to be rapid and adequate. Patients with long-term ICI treatment could have durable survival benefits even if the ICI was discontinued due to toxicity. However, this is only a case report and the results need to be further explored with larger samples.

## Data Availability Statement

The original contributions presented in the study are included in the article/**Supplementary Material**. Further inquiries can be directed to the corresponding author.

## Ethics Statement

Written informed consent was obtained from the individual(s) for the publication of any potentially identifiable images or data included in this article.

## Author Contributions

ZY and GZ: data collection and manuscript writing. QS, JS, and ML: data collection. SJ: project development and manuscript writing. All authors contributed to the article and approved the submitted version.

## Funding

This work was supported by Major special projects funds of the 13th five-year plan (2018ZX09201013): The construction of international standard clinical evaluation platform of novel drugs for the prevention of major disease.

## Conflict of Interest

The authors declare that the research was conducted in the absence of any commercial or financial relationships that could be construed as a potential conflict of interest.
